# In Situ Gel Formation, Pore Network Evolution and Mechanical Degradation of Red Sandstone Under Chemical–Wet–Dry Cycles

**DOI:** 10.3390/gels12060499

**Published:** 2026-06-04

**Authors:** Jingjing Zhang, Ning Liang, Dingli Su

**Affiliations:** 1 School of Civil Engineering and Architecture, Guangxi University of Science and Technology, Liuzhou 545006, China; 20240301014@stdmail.gxust.edu.cn; 2 School of Civil and Transportation Engineering, Guangdong University of Technology, Guangzhou 510006, China; dingli_su@163.com; 3 Guangzhou Jianyan Engineering Technology Co., Ltd., Guangzhou 510440, China; 4 Guangzhou Construction Engineering Co., Ltd., Guangzhou 510288, China

**Keywords:** in situ hydrogel, red sandstone, chemical–wet–dry cycles, multiscale damage, statistical constitutive model

## Abstract

This study investigates in situ hydrogel formation and its regulating effect on multiscale damage evolution in red sandstone subjected to chemical–wet–dry cycles. Uniaxial compression, X-ray diffraction, scanning electron microscopy coupled with energy-dispersive spectroscopy, mercury intrusion porosimetry, and inductively coupled plasma mass spectrometry tests were performed to characterize mechanical degradation, mineral alteration, pore-network evolution, ion migration, and gel micromorphology. By combining multiscale experimental characterization with a segmented statistical damage constitutive model, this study describes the hydrogel-mediated damage evolution of red sandstone under chemical–wet–dry cycles. The mechanical properties of red sandstone show nonlinear degradation, with a deterioration order of acidic > alkaline > neutral, and this effect intensifies with increasing cycle number. After 15 cycles at pH = 3, the compressive strength and elastic modulus decreased by 38.21% and 27.12%, respectively. Both acidic and alkaline environments promoted pore development in red sandstone. After 15 cycles at pH = 3, the porosity increased from 21.51% to 24.51%, and the most probable pore diameter shifted from 21.32 μm to 25.88 μm. The porosity increased by 2.86% at pH = 11, and in situ hydrogels formed under alkaline conditions partially filled pores and inhibited crack propagation. The developed model effectively reproduced the mechanical evolution of red sandstone, with all fitted results showing *R*^2^ values no lower than 0.92. These findings provide a basis for evaluating hydrogel-regulated damage in red sandstone and support the application of in situ gel materials in geotechnical engineering.

## 1. Introduction

Driven by the rapid expansion of the global economy, environmental pollution has significantly intensified. Complex hydrochemical environments have become a critical concern in geotechnical engineering, especially for the in situ application of gel materials [1]. Specifically, fossil fuel combustion releases large amounts of acidic gases, which form acid rain through atmospheric reactions. Industrial effluents also frequently alter the chemical composition of groundwater systems [2]. Beyond these anthropogenic influences, natural factors also alter environmental chemical properties. These changes pose major risks to the structural integrity and safety of geotechnical engineering works [3,4]. Red sandstone is widely encountered in geotechnical engineering, especially in the construction of subgrades, tunnels, and slopes [5]. However, soft red sandstone has inherent low strength, weak intergranular cementation, and a strong softening tendency after long-term water exposure. Under complex hydrochemical conditions, chemical reactions in this rock not only degrade its mechanical properties [6,7], but may also promote the in situ formation of hydrogel materials. In addition, periodic fluctuations in groundwater and river levels subject rock masses to repeated wet–dry cycles. These cycles induce internal damage in the rock and may also affect the formation and stability of in situ hydrogels, thereby threatening the long-term stability of engineering structures.

Extensive experimental evidence has demonstrated that chemical erosion leads to the simultaneous degradation of both the macrostructure and microstructure of rocks. This deterioration typically manifests as reductions in the elastic modulus, peak strength, P-wave velocity, and internal friction angle [8,9,10,11]. It also results in measurable increases in permeability, porosity, water absorption, and mass loss rate [12,13,14]. These changes in pore structure and chemical environment may further affect the nucleation and growth of in situ hydrogels in the rock matrix. The synergistic effect of physical and chemical processes is widely recognized as the main driver of rock degradation [15]. Physical mechanisms generally involve mineral expansion–contraction and softening, whereas chemical processes include mineral dissolution, hydrolysis, ion exchange, and the formation of secondary minerals [16]. These coupled processes facilitate the initiation and subsequent propagation of microcracks. Hydrochemical solutions can profoundly alter mineralogical composition, microstructural frameworks, ionic concentrations, and permeability, thereby accelerating the evolution of internal damage [17,18]. Variations in solution chemistry not only affect compressive strength and the elastic modulus [19] but also exert pronounced effects on rock microstructure [20,21,22]. Additionally, the hydrochemical environment significantly influences rock deformation behavior. As solution acidity increases, macroscopic mechanical properties tend to decline, whereas plastic deformation capacity and ductility rise, resulting in increasingly complex damage mechanisms [23,24]. Therefore, rock failure should be regarded as the cumulative result of coupled physical, chemical, and mechanical processes rather than the consequence of a single controlling factor. Studies focusing on only one variable may not fully reflect the complex service conditions encountered in practical engineering, especially under chemical–wet–dry cyclic environments associated with in situ gel formation.

Ensuring the engineering stability of rocks under diverse environmental and loading conditions necessitates the development of reliable constitutive models that account for these multiple factors. Statistical damage mechanics provides a useful framework for constructing damage variables based on probabilistic strength distributions of mesoscopic rock elements, enabling the quantitative tracking of damage progression [7]. In recent years, various constitutive models have been proposed to address damage induced by chemical action or wet–dry cycles [25,26,27,28]. However, research on rock constitutive models has primarily examined single-factor or dual-factor coupling effects, such as chemical–mechanical or wet–dry cycle–mechanical models [29,30,31,32]. Studies remain limited on the combined effects of the chemical–wet–dry cycle interaction and external loading. Furthermore, the quantification of damage from chemical and wet–dry cycles often relies on macroscopic mechanical parameters, such as the elastic modulus, fractal dimension, and energy dissipation [21,32,33]. These methods cannot reveal the mechanism by which in situ hydrogel formation influences rock damage at the mesoscopic scale. By contrast, defining damage variables based on microstructural features offers a more effective approach to identifying the underlying mechanisms of rock deterioration [25]. This study investigates the influence of the chemical environment and wet–dry cycles on in situ hydrogel formation, internal structure evolution, and load-bearing and deformation behavior of red sandstone. Specifically, it analyzes the damage evolution process of red sandstone, and establishes a statistical damage constitutive model considering the hydrogel-mediated effect.

Despite the above advances, existing research still has three key limitations: First, most studies focus on single or dual coupling effects and rarely consider the simultaneous effects of the chemical environment, wet-dry cycles, and external loading, especially overlooking the in situ formation of hydrogels under these coupling conditions. Second, damage quantification is highly dependent on macroscopic mechanical parameters. The multiscale correlation between ion migration, mineral alteration, pore network evolution, macroscopic mechanical deterioration, and in situ hydrogel formation has not been systematically revealed. Third, the existing statistical damage constitutive models do not systematically integrate the coupled effects of pH value and cycle number, and do not consider the regulating effect of in situ hydrogels on rock damage. This limits the accurate prediction of the deformation and failure of red sandstone in complex hydrochemical environments and also restricts the engineering application of in situ gel materials. This study uses soft red sandstone as the research material to fill the above gaps. We carried out uniaxial compression tests under different pH conditions and cycle numbers, combined with multiscale analysis methods including X-ray diffraction (XRD), scanning electron microscopy with energy dispersive spectroscopy (SEM-EDS), mercury intrusion porosimetry (MIP), and inductively coupled plasma mass spectrometry (ICP-MS). Full experimental details are provided to ensure the reproducibility of all test results. This comprehensive method systematically reveals the mechanism of in situ hydrogel formation and the multiscale damage evolution of red sandstone under chemical–wet–dry cycles. On this basis, a segmented statistical damage constitutive model incorporating the coupled effects of the chemical environment, wet–dry cycles, compressive loading and the hydrogel regulation effect is established.

## 2. Results and Discussion

### 2.1. Mechanical Evolution of Red Sandstone Under Chemical–Wet–Dry Interaction

#### 2.1.1. Mass Loss and P-Wave Velocity

**Figure 1:**
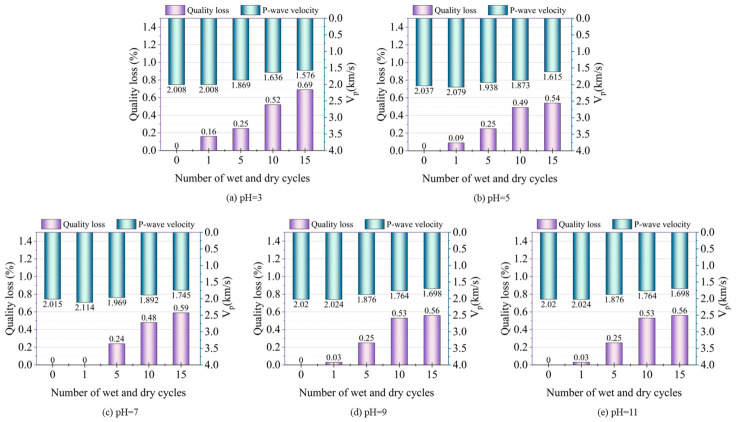
Progression of mass loss and P-wave velocity in red sandstone across successive wet–dry cycles.

As illustrated in Figure 1, the mass loss of the specimens increased progressively with the number of wet–dry cycles owing to chemical interactions. This phenomenon is primarily attributed to mineral dissolution, which leads to the continuous detachment of framework particles and the release of active ions involved in in situ hydrogel formation. Consequently, mass loss gradually accumulates with successive wet–dry cycles.

The P-wave velocity exhibited complex behavior: it increased slightly during early wet–dry cycles under acidic and alkaline conditions but decreased continuously under neutral conditions. The magnitude of this decrease varied significantly across different hydrochemical environments. Specifically, the most substantial decrease in P-wave velocity occurred at pH = 3 after 15 wet–dry cycles, with a reduction of 0.43 km/s, whereas the smallest decrease (0.27 km/s) was observed at pH = 7. This initial increase in velocity suggests that mineral hydration and subsequent expansion may partially fill internal pores [34], while the in situ hydrogels formed under alkaline conditions may further enhance this pore-filling effect. However, the cumulative effect of ongoing mineral dissolution and particle detachment, coupled with the progressive evolution of the pore structure, eventually results in a consistent decrease in P-wave velocity.

#### 2.1.2. Mechanical Properties

**Figure 2:**
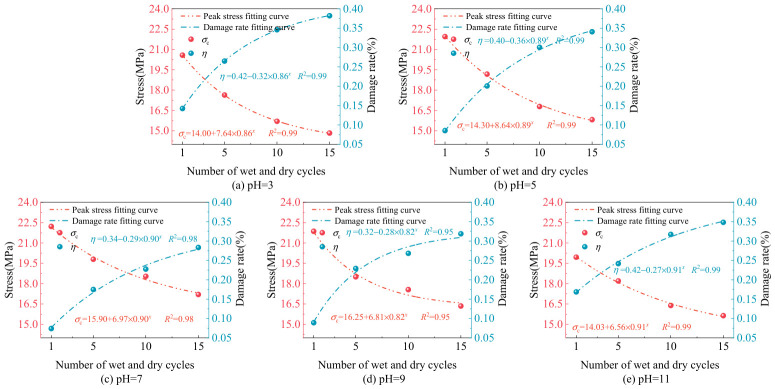
Evolution of peak compressive strength and corresponding degradation degree under chemical–wet–dry interactions.

**Figure 3:**
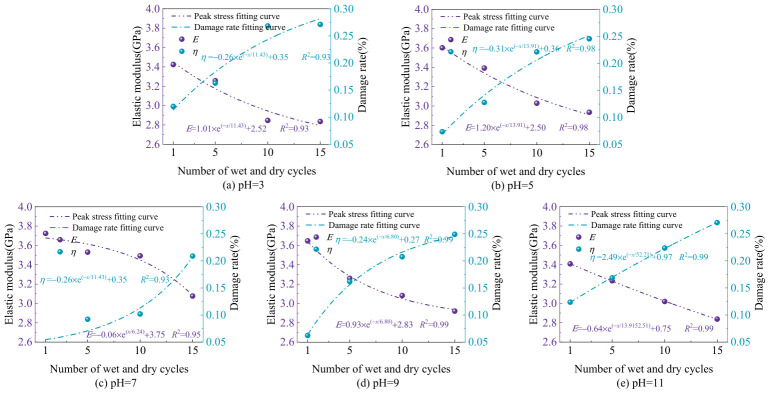
Variations in elastic modulus and degradation degree as a function of cycle frequency across different hydrochemical environments.

The evolutions of peak strength ($\sigma_{c}$), elastic modulus (E), and degradation degree (η) for red sandstone samples under chemical–wet–dry interactions are presented in Figure 2 and Figure 3. The fitting results indicate that these parameters effectively describe the mechanical variations with increasing cycle number under different pH conditions. The degradation degree (η) is calculated as follows:

(1) $$\eta =\frac{J_{0}-J}{J_{0}}$$

where $J_{0}$ and J represent the mechanical properties of the intact red sandstone and the corresponding values measured after chemical–wet–dry interactions, respectively.

The peak strength was significantly influenced by the synergy between the chemical environment and wet–dry cycles, as well as the regulating effect of in situ hydrogels formed under alkaline conditions. After 15 cycles, the degradation degree of peak strength varied under different pH conditions: η=38.21% (pH = 3), η=34.08% (pH = 5), η=28.33% (pH = 7), η=31.88% (pH = 9), and η=34.83% (pH = 11). Interestingly, at *N* = 1, the degradation degree at pH = 11 (η=16.88%) was higher than that at pH = 3 (η=14.00%). However, with prolonged cycling, acidic conditions ultimately exerted a more pronounced effect on mechanical integrity, whereas in situ hydrogels formed under alkaline conditions may have partially mitigated strength degradation.

As shown in Figure 3, the elastic modulus exhibited a nonlinear decline with increasing wet–dry cycles. Taking pH = 7 as a representative case, the degradation degrees for the elastic modulus were 4.24% (*N* = 1), 9.23% (*N* = 5), 10.21% (*N* = 10), and 20.90% (*N* = 15). For an equivalent number of cycles, the elastic modulus values followed the order: neutral > alkaline > acidic. For instance, at *N* = 10, the modulus values were 2.85 GPa (pH = 3), 3.03 GPa (pH = 5), 3.53 GPa (pH = 7), 3.08 GPa (pH = 9), and 3.02 GPa (pH = 11). The smaller modulus reduction in alkaline environments is closely related to the supporting effect of in situ hydrogels in rock pores.

**Figure 4:**
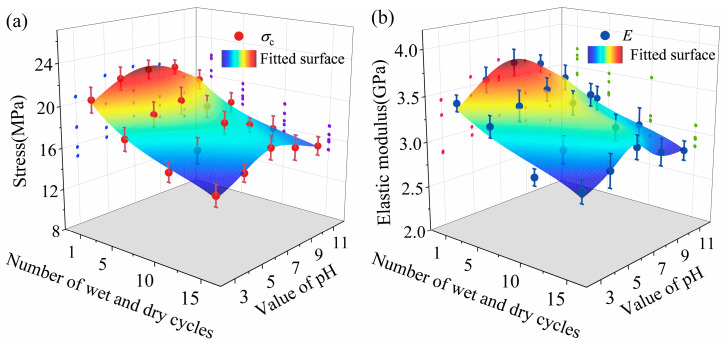
Three-dimensional fitted surfaces representing the coupled influence of pH and cycle frequency on mechanical parameters: (**a**) peak strength; (**b**) elastic modulus.

Figure 4 and Table 1 summarize the impact of chemical–wet–dry cycles on these parameters. Notably, wet–dry cycles exerted a stronger influence on the mechanical properties of the rock specimens than the solution pH. Empirical relationships were formulated to quantify these interactions, as expressed in Equations (2) and (3) and visualized through the fitted surfaces in Figure 4. The coefficient of determination (R^2^) values for peak strength and elastic modulus were 0.98 and 0.92, respectively, reflecting strong agreement between the experimental data and the fitting results. These empirical relationships provide a quantitative basis for the subsequent constitutive model incorporating hydrogel-mediated effects.

(2) $$\sigma_{c}=\frac{18.46-0.64x-2.02y+0.17y^{2}-0.0007y^{3}}{1+0.0004x-0.0025x^{2}-0.0006x^{3}-0.15y+0.01y^{2}}\;R^{2}=0.90$$

(3) $$E=\frac{30.57-0.83x-4.60y+0.29y^{2}-0.0032y^{3}}{1-0.01x-0.0010x^{2}-0.00002x^{3}-0.01y+0.01y^{2}}\;R^{2}=0.92$$

where x denotes the number of wet–dry cycles and y denotes the pH.

### 2.2. Impact of Chemical–Wet–Dry Interaction on the Microstructure of Red Sandstone

#### 2.2.1. Ion Interactions

**Figure 5:**
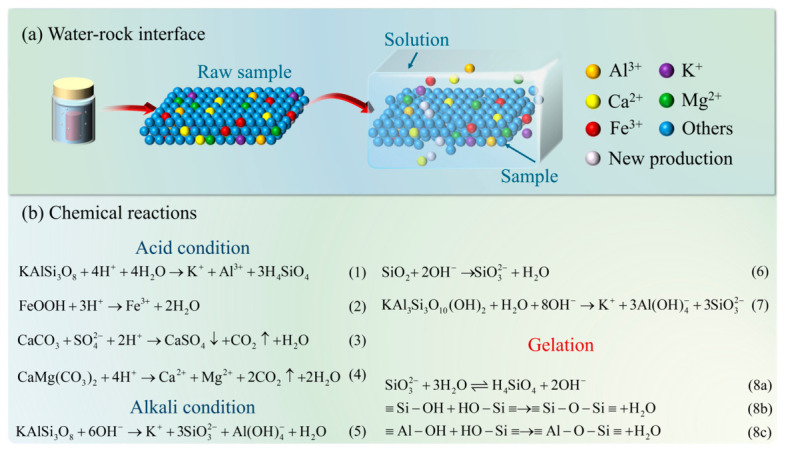
Conceptual mechanism illustrating ionic interactions at the water–rock interface and subsequent chemical reactions.

Ion interactions within the red sandstone are fundamental to microstructural damage, as illustrated in Figure 5.

**Figure 6:**
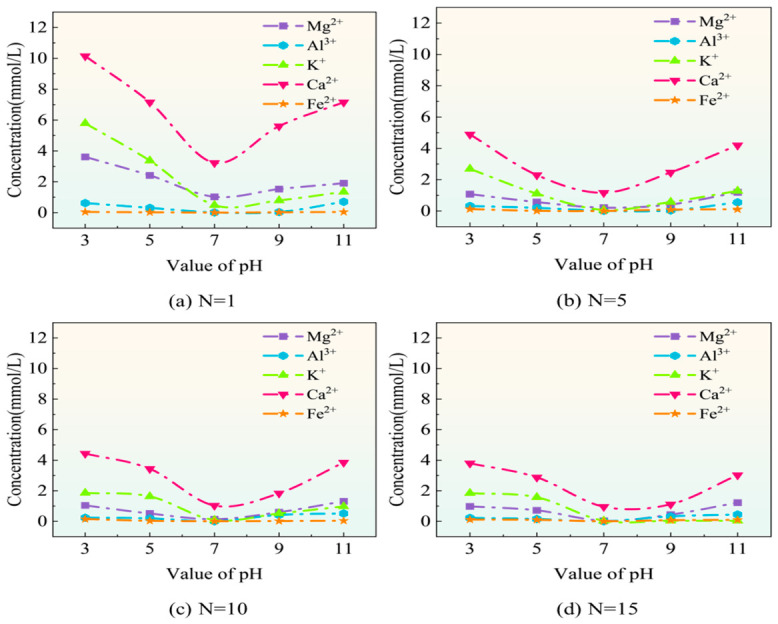
Dynamic evolution of ion concentrations within the soaking solution during cyclic interactions.

Variations in the ionic concentrations of Mg^2+^, Al^3+^, K^+^, Ca^2+^, and Fe^2+^ in the soaking solution were analyzed using ICP-MS (Figure 6). These ions are the key precursors for the in situ formation of hydrogels under alkaline conditions. Generally, ion concentrations decreased an increasing number of cycles, with distinct variations observed across different pH levels.

Specifically, the Ca^2+^ concentration typically declined as cycles progressed. At pH = 3, the Ca^2+^ concentration reached 10.15 mmol/L after one cycle but decreased by 52%, 54%, and 63% after 5, 10, and 15 cycles, respectively. By contrast, at pH = 7, the concentration varied only slightly, ranging from 3.225 mmol/L (*N* = 1) to 0.957 mmol/L (*N* = 15). This indicates that Ca^2+^ leaching is significantly more pronounced under acidic conditions. Similarly, K^+^ concentrations exhibited high sensitivity to acidic environments; at *N* = 1, the K^+^ concentration at pH = 3 (5.79 mmol/L) was markedly higher than that at pH = 7 (0.48 mmol/L), further confirming that acidity promotes the release of K^+^. The migration and release of these active ions provide a chemical basis for subsequent water–rock reactions and the possible gelation process in alkaline environments.

**Figure 7:**
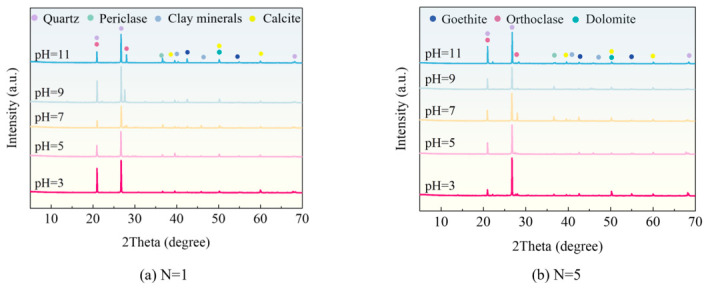

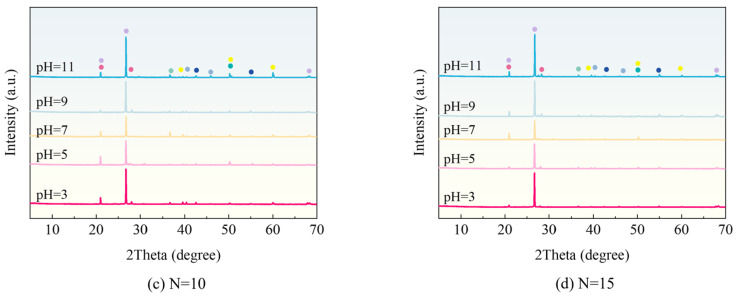
Comparative X-ray diffraction patterns of red sandstone specimens following exposure to different hydrochemical environments.

XRD analysis was performed to evaluate the alterations in mineralogical composition following chemical–wet–dry cycles, with the resulting diffraction patterns across various hydrochemical conditions presented in Figure 7. These mineralogical changes influence the release of gel-forming precursors and the evolution of pore space for in situ hydrogel growth. While the primary mineral diffraction peaks remained detectable, their intensities exhibited notable variations driven by the specific hydrochemical environment and the cumulative impact of the wet–dry cycles. In these patterns, an increase in the diffraction peak intensity of a mineral signifies a higher relative content, whereas a decrease indicates mineral dissolution [35,36]. Specifically, quartz peak intensity rose with increasing wet–dry cycles, whereas the intensity of other soluble minerals decreased. This reduction is attributed to dissolution processes that facilitate pore formation, weaken interparticle cementation, and induce microcracking, thereby increasing the water–rock contact area and providing more space for in situ hydrogel growth.

**Figure 8:**
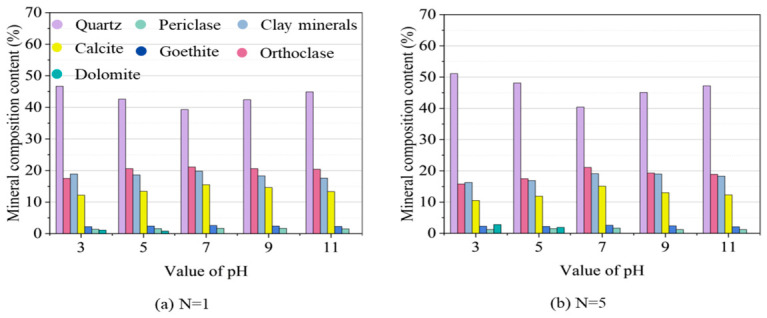

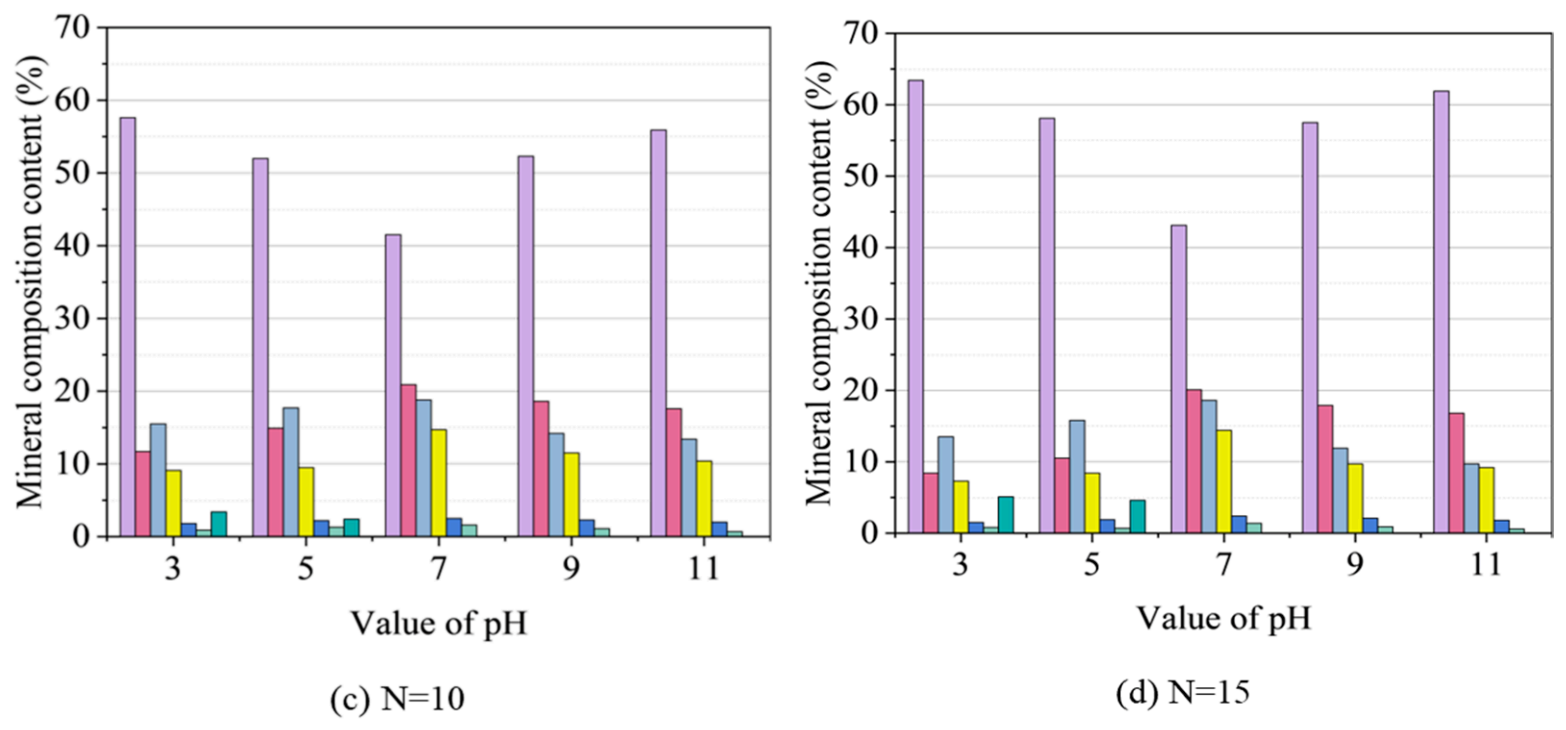
Mineralogical composition evolution of red sandstone relative to solution pH and the number of chemical–wet–dry cycles.

As illustrated in Figure 8, the variations in mineral content after chemical–wet–dry interactions were systematically analyzed. The initial mineralogical framework of the specimens was dominated by quartz (38.2%), feldspar (21.8%), and clay minerals (20.2%). Although the types of major minerals remained consistent across samples, their relative contents shifted slightly following treatment, with a similar overall trend observed across the different hydrochemical solutions.

As the number of cycles increased, soluble minerals underwent continuous physicochemical reactions with the aqueous solutions, resulting in measurable pore expansion and crack development. Upon reaching 15 cycles, the quartz content across the various hydrochemical environments was recorded at 63.4%, 58.1%, 43.1%, 57.5%, and 61.9% for the respective pH levels. Under acidic conditions, calcium sulfate was detected, primarily resulting from the chemical reaction between Ca^2+^—released via calcite dissolution—and SO_4_^2−^ from the sulfuric acid solution, leading to CaSO_4_ precipitation. By contrast, specimens subjected to neutral conditions exhibited relative stability, with mineral content variations remaining below 5%, indicating a comparatively weak chemical effect. Overall, the increasing frequency of wet–dry cycles led to a reduction in soluble minerals, such as feldspar and clay, owing to irreversible water–rock reactions that caused significant mineral damage and content loss. In alkaline environments, the active silicon and aluminum components released from these soluble minerals are the key components for forming the formation of the three-dimensional network structure of in situ hydrogels.

#### 2.2.2. Pore-Size Distribution and Porosity

**Figure 9:**
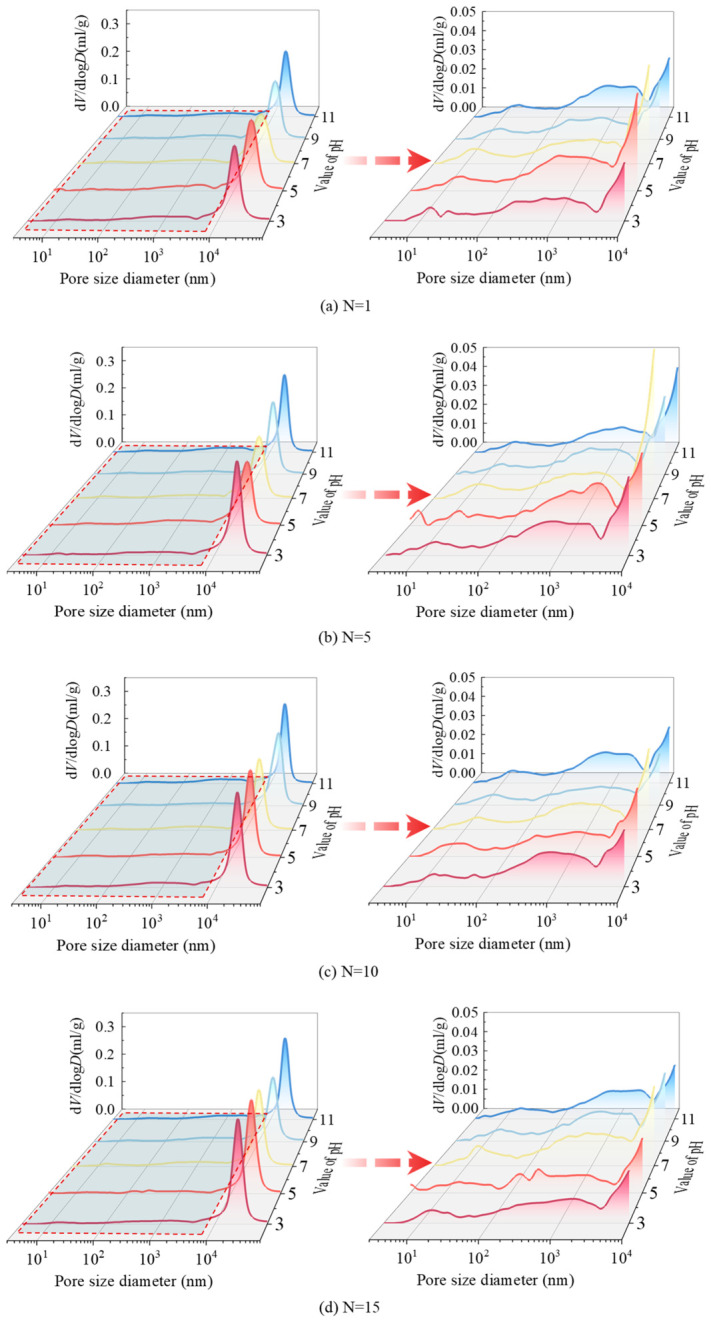
Log-differential mercury intrusion curves detailing shifts in pore-size distribution under chemical–wet–dry cycle interaction.

The impact of chemical–wet–dry cycles on the internal pore structure of the red sandstone was further investigated using MIP tests. The evolution of pore structure not only determines the mechanical properties of the rock but also may influence the available gelation space and distribution characteristics of in situ hydrogels. Figure 9 illustrates the relationship between pore diameter and pore volume increase resulting from these interactions, where the vertical axis represents the pore volume increase per log pore-size interval (d*V*/dlg*D*, where *V* and *D* denote pore volume and diameter, respectively). The results indicate that wet–dry cycling primarily increases the number of pores while exerting a minimal influence on the overall pore-size scale. The dominant pore diameters were concentrated between 10 and 30 μm, falling within the super-macropore interval. This structural characteristic is closely linked to the inherent properties of soft red sandstone, which is characterized by weak interparticle cementation and well-developed primary porosity. For this analysis, the pore structure is categorized into five distinct groups based on size [37]: super-macropores (>10 μm), macropores (2.5–10 μm), mesopores (0.5–2.5 μm), micropores (0.1–0.5 μm), and ultra-micropores (<0.1 μm).

In the natural, untreated state, the most probable pore diameter of the red sandstone was 21.32 μm, placing it within the super-macropore range. Under neutral conditions (pH = 7), increasing the number of wet–dry cycles (*N*) caused the peak value of the distribution curve to rise, while the position of the most probable pore diameter remained relatively unchanged (Figure 9). This indicates that under neutral cycling, the primary effect is an increase in the number of pores, rather than a shift in pore scale. Conversely, as the acidity of the solution increased, the most probable pore diameter exhibited a distinct shift toward the larger super-macropore range. Specifically, rightward shifts of 4.56 μm and 2.84 μm were recorded at pH = 3 and pH = 5, respectively, relative to the natural state (21.32 μm), highlighting the intensified pore expansion triggered by acidic environments. Under alkaline conditions, small pores evolved into larger voids, thereby broadening the pore distribution range and enhancing internal connectivity, while the in situ hydrogels formed under alkaline conditions partially fill the pores and regulate the evolution of the pore network.

The progression of wet–dry cycles (*N*) led to higher cumulative mercury intrusion across all specimens, reflecting a clear upward trend in porosity, as detailed in Table 2. At pH = 7, porosity increased from an initial 21.51% to 23.14% after cycling. This increase was significantly more pronounced under extreme pH conditions; specifically, the porosity increments reached 3.00% at pH = 3 and 2.86% at pH = 11. The slightly lower porosity increment under alkaline conditions can be attributed to the pore-filling effect of in situ hydrogels formed within the rock matrix.

### 2.3. Damage Constitutive Model

#### 2.3.1. Damage Variable

According to damage mechanics theory, the initiation and subsequent propagation of microcracks within rock masses—driven by either external loading or environmental stressors—lead to significant internal damage accumulation and eventual macroscopic failure [38]. For red sandstone subjected to chemical–wet–dry cycle interactions, the overall damage evolution can be categorized into three primary components [39]: chemical ($D_{C}$), wet–dry cycle ($D_{N}$), and loading ($D_{S}$). Chemical damage ($D_{C}$) primarily originates from pore expansion induced by mineral dissolution, whereas wet–dry cycle damage ($D_{N}$) is associated with the nonuniform expansion and contraction of mineral particles under repeated wet–dry cycles and the capillary pressure effect generated by water infiltration [40]. Both damage types result in microstructural deterioration, increased porosity, and degradation of mechanical properties, while in situ hydrogels can partially offset these damage effects via pore-filling effects. Loading damage ($D_{S}$) characterizes the progressive growth and linkage of internal microcracks along the stress axis, and this process may also be partially inhibited by in situ hydrogels.

**Figure 10:**
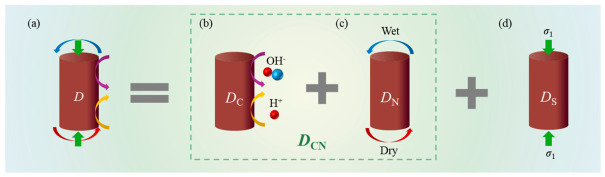
Schematic representation of damage evolution components under coupled chemical–wet–dry cycle and axial loading conditions: (**a**) total damage; (**b**) chemical damage; (**c**) wet–dry cycle damage; (**d**) loading damage.

Using Lemaitre’s strain equivalence hypothesis [41] and considering the distinct damage states illustrated in Figure 10b,c, the constitutive relationship for red sandstone after chemical–wet–dry cycling is expressed as:

(4) $$\sigma_{CN}^{*}=E\left(1-D_{CN}\right)\varepsilon_{CN}$$

where $\sigma_{CN}^{*}$ represents the effective stress after chemical–wet–dry cycling, and E is the elastic modulus.

The damage constitutive equation of red sandstone, considering the combined effects of chemical–wet–dry cycle interaction and axial loading, can be expressed as [39]:

(5) $$\sigma =E\left(1-D_{CN}\right)\left(1-D_{S}\right)\varepsilon$$

Combining Equations (4) and (5), the total damage variable (*D*) is defined as:

(6) $$D=D_{CN}+D_{S}-D_{CN}D_{S}$$

(1) Chemical–Wet–Dry Cycle–Coupled Damage

Chemical erosion results from the interaction between rock minerals and aggressive solutions, causing progressive mineralogical degradation and releasing active ions involved in in situ hydrogel formation. Simultaneously, wet–dry cycles initiate microcracking owing to repeated physical stresses, leading to diverse spatiotemporal damage patterns. The resulting rock degradation is significantly influenced by solution diffusion and reaction kinetics, which also influence the gelation process of in situ hydrogels. While prior research has often quantified damage using macroscopic parameters such as peak strain, elastic modulus, or residual strength [13,21,27], defining damage variables based on porosity evolution provides a more fundamental understanding of internal structural reorganization and hydrogel pore-filling effects. Accordingly, a porosity-based coupled damage variable is expressed as:

(7) $$D_{CN}=1-\frac{f_{CN}}{f_{0}}$$

where $f_{0}$ represents the mechanical parameters of the specimen in its initial, intact state, whereas $f_{CN}$ and $f_{N}$ represent the parameters measured after *N* wet–dry cycles in a solution with a specific pH of C.

The interaction between chemical stressors and wet–dry cycles induces intricate physicochemical reactions among rock particles, ultimately driving internal structural reorganization and the evolution of porosity, which may also influence the available pore space for in situ hydrogel growth. Because changes in porosity are recognized as a fundamental cause of macroscopic mechanical degradation, porosity-based damage variables have been widely used in related studies to quantify damage resulting from chemical–wet–dry cycle interactions [39,42]. Such variables can also reflect the pore-filling effect of hydrogels.

**Figure 11:**
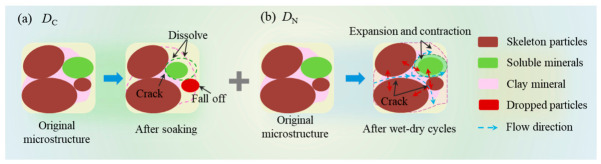
Microscopic conceptualization of coupled damage mechanism: (**a**) chemical-induced dissolution; (**b**) wet–dry cycle-induced expansion and contraction.

Accordingly, the chemical–wet–dry cycle–coupled damage variable can be expressed as:

(8) $$D_{CN}=\frac{n_{CN}-n_{0}}{1-n_{0}}$$

where $D_{C}$ and $D_{N}$ denote the chemical damage and the wet–dry cycle damage, respectively. In this context, $n_{CN}$ and $n_{N}$ represent the porosity of the specimen after undergoing *N* wet–dry cycles in a solution with a pH of C, whereas $n_{0}$ denotes the initial porosity of the specimen. The conceptual mechanisms for both chemical and wet–dry cycle damage are illustrated in Figure 11.

Porosity is defined as the ratio of pore volume to total volume. Damage refers to the accumulation of irreversible microstructural defects caused by external forces and environmental factors. This damage can be partially mitigated by in situ hydrogels. Damage is often characterized by the reduction in the effective load-bearing area relative to the initial load-bearing area. Consequently, using Equation (8) directly to quantify chemical–wet–dry cycle coupled damage has limitations, as it does not consider the hydrogel-mediated pore-filling effect.

Lemaitre’s strain-equivalence principle posits that the damage variable should be defined by considering the role of the effective load-bearing area. This relationship can be formally expressed as follows:

(9) $$D_{CN}=\frac{\Delta S^{d}}{{\left(S^{d}\right)}_{0}}=\left(\frac{\Delta r^{d}}{{\left(r^{d}\right)}_{0}}\right)={\left(\frac{\Delta V^{d}}{{\left(V^{d}\right)}_{0}}\right)}^{\frac{2}{3}}$$

where $\Delta S^{d}$ and ${\left(S^{d}\right)}_{0}$ represent the effective load-bearing areas for the damaged and original states, respectively; $\Delta r^{d}$ and ${\left(r^{d}\right)}_{0}$ denote the effective load-bearing radii for the damaged and initial configurations, respectively; and $\Delta V^{d}$ and ${\left(V^{d}\right)}_{0}$ indicate the effective load-bearing volumes under the damaged and original conditions, respectively.

Building upon this foundation, Li et al. [42] further defined the following refined porosity-based damage variable:

(10) $$D_{CN}={\left(\frac{n_{CN}-n_{0}}{1-n_{0}}\right)}^{\frac{2}{3}}$$

(2) Loading Damage

Rock is inherently heterogeneous, and its microscopic heterogeneity primarily stems from structural defects such as pores, microcracks, and variations in mineral composition, as well as the distribution of in situ hydrogels. Under external loading, these spatially random defects cause damage to initiate at localized weak points, which subsequently propagates through the rock mass, while hydrogels can inhibit this crack propagation. The Weibull distribution is widely used to characterize this probabilistic damage behavior, as it effectively describes the failure probability distribution of microelements arising from heterogeneous microstructures and strength dispersion, while allowing the regulating effect of hydrogels to be considered.

The strength distribution of rock microelements is commonly modeled using the following Weibull distribution, providing a statistical representation of strength variability:

(11) $$P\left(F\right)=\frac{m}{F_{0}}{\left(\frac{F}{F_{0}}\right)}^{m-1}\exp\left[-{\left(\frac{F}{F_{0}}\right)}^{m}\right]\;F\geq 0$$

where F denotes the strength of a rock micro-element, and m and $F_{0}$ represent the Weibull shape and scale parameters, respectively.

Let $S_{f}$ denote the number of failed micro-elements and S represent the total number of micro-elements within the sample. Under axial loading conditions, the loading damage variable can be defined as follows:

(12) $$D_{S}=\frac{S_{f}}{S}$$

When the micro-element strength falls within the interval $\left[F,F-dF\right]$, the number of damaged micro-elements is given by $SP\left(F\right)dF$. By considering the cumulative number of failed micro-elements with strength thresholds ranging from 0 to F, and substituting this into Equation (12), the total loading damage variable is obtained as follows:

(13) $$D_{S}==\frac{S\int_{0}^{F}\frac{m}{F_{0}}{\left(\frac{x}{F_{0}}\right)}^{m-1}\exp\left[-{\left(\frac{x}{F_{0}}\right)}^{m}\right]dx}{N}=1-\exp\left[1-{\left(\frac{F}{F_{0}}\right)}^{m}\right]$$

The rock micro-element strength is evaluated using the Mohr–Coulomb criterion as follows:

(14) $$F=f\left(\sigma^{*}\right)=\sigma^{*}-\tan^{2}\left(45^{\circ }+\varphi /2\right)\sigma_{3}^{*}$$

(15) $$\sigma_{i}^{*}=\frac{\sigma_{i}}{1-D}$$

where $\sigma^{*}$, $\sigma_{1}^{*}$, and $\sigma_{3}^{*}$ denote the effective, maximum effective principal, and minimum effective principal stresses, respectively. The parameter φ represents the internal friction angle of the sample.

By integrating Equations (14) and (15), the following expression is obtained:

(16) $$1-D=\left[\sigma_{1}-\tan^{2}(45^{\circ }+\varphi /2)\sigma_{3}\right]/F$$

This criterion is based on the assumption that microelements obey Hooke’s law and Lemaitre’s strain-equivalence principle. Under these conditions, the principal strain $\varepsilon_{1}$ linked to the principal stress $\sigma_{1}$ can be expressed as follows:

(17) $$\varepsilon_{1}=\left[\sigma_{1}^{*}-v\left(\sigma_{2}^{*}+\sigma_{3}^{*}\right)\right]/E=\left[\sigma_{1}-v\left(\sigma_{2}+\sigma_{3}\right)\right]/\left[\left(1-D\right)E\right]$$

where ν denotes the Poisson’s ratio of the rock sample.

By rearranging this relationship, Equation (9) can be rewritten as:

(18) $$1-D=\left[\sigma_{1}-v\left(\sigma_{2}+\sigma_{3}\right)\right]/E\varepsilon_{1}$$

By further combining Equations (16) and (18), the expression for F is derived as:

(19) $$F=\frac{E\varepsilon_{1}\left[\sigma_{1}-\tan^{2}\left(45^{\circ }+\varphi /2\right)\sigma_{3}\right]}{\sigma_{1}-\nu \left(\sigma_{2}+\sigma_{3}\right)}$$

Because axial compressive strength tests are adopted in this study, $\sigma_{2}=\sigma_{3}=0$. By combining Equations (13) and (19), the loading damage variable $D_{S}$ for red sandstone during uniaxial compression under chemical–wet–dry cycle interaction can be derived as follows:

(20) $$D_{S}=1-\exp\left[-{\left(\frac{E\varepsilon_{1}}{F_{0}}\right)}^{m}\right]$$

The peak-point criterion was used for parameter identification in the Weibull model, following established research methodologies [43,44,45]. At the peak of the stress–strain curve, the first derivative of stress with respect to strain is zero, establishing the following critical relationship:

(21) $$\left.\frac{\partial \sigma_{1}}{\partial \varepsilon_{1}}\right|(\varepsilon_{1}=\varepsilon_{p},\sigma_{1}=\sigma_{p})=0$$

where $\varepsilon_{p}$ and $\sigma_{p}$ denote the peak strain and stress, respectively, and $\sigma_{1}$ represents the first principal stress.

The constitutive model integrates a microcrack closure effect to represent the rock’s nonlinear mechanical behavior during the initial compaction phase:

(22) $$\sigma_{i}=\left\{\begin{array}{l} E\exp\left[-{\left(\frac{E\varepsilon_{1}}{F_{0}}\right)}^{m}\right]\left(\varepsilon_{1}-\varepsilon_{1cc}\right)\;\left(\varepsilon_{1}\geq \varepsilon_{1cc}\right)\\ E\exp\left[-{\left(\frac{E\varepsilon_{1}\left(\varepsilon_{p}-\varepsilon_{1cc}\right)}{F_{0}}\right)}^{m}\right]\left(\varepsilon_{p}-\varepsilon_{1cc}\right)+\sigma_{1cc} \end{array}\right.$$

where $\varepsilon_{1cc}$ denotes the microcrack closure strain, and $\sigma_{1cc}$ represents the microcrack closure stress.

Combining Equations (21) and (22) leads to the following derivations for the model parameters:

(23) $$m=\frac{1}{\ln\left(\frac{E\left(\varepsilon_{p}-\varepsilon_{1cc}\right)}{\sigma_{p}-\sigma_{1cc}}\right)}$$

(24) $$F_{0}=E\left(\varepsilon_{p}-\varepsilon_{1cc}\right)m^{\frac{1}{m}}$$

#### 2.3.2. Formulation of the Damage Constitutive Model

(1) Division of Damage Evolution Stages

**Figure 12:**
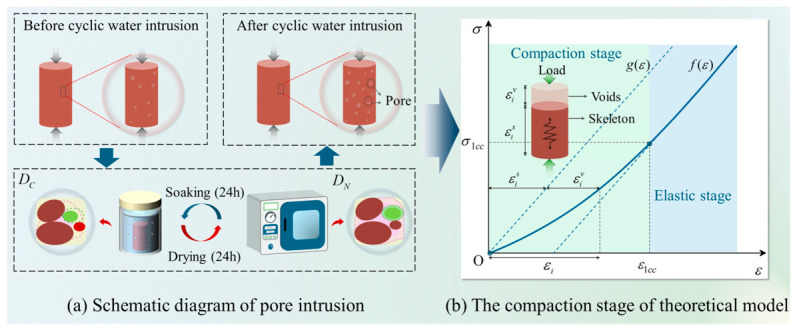
Theoretical framework for pore strain evolution: (**a**) pore intrusion schematic and (**b**) the compaction phase within the constitutive model.

As illustrated in Figure 12a, the interaction between chemical stressors and wet–dry cycles promotes the expansion of primary pores and the initiation of new microcracks within the red sandstone, resulting in a significant increase in both pore quantity and total volume, while in situ hydrogels formed under alkaline conditions partially fill pores. Deformation under axial loading can be divided into two primary stages: an initial compaction phase involving irreversible deformation driven by the closure of pores and microcracks, which is significantly affected by hydrogel filling, followed by a stage governed by the solid mineral skeleton. This secondary stage encompasses linear-elastic deformation before yielding and nonlinear plastic deformation thereafter, as depicted in Figure 12b. Because these two mechanisms exhibit fundamental distinctions in their dominant structures, deformation characteristics, and mechanical responses, they correspond to unique modes of damage evolution. Consequently, a piecewise constitutive model considering hydrogel-mediated effects is required to better represent these distinct mechanical behaviors.

The total deformation of the specimen under axial stress is primarily governed by two mechanisms: micropore compaction and the stress-induced deformation of the mineral skeleton. This relationship is expressed as:

(25) $$\varepsilon_{i}=\varepsilon_{i}^{v}+\varepsilon_{i}^{s}$$

where $\Delta l_{i}$ denotes the total deformation of the sample; $\Delta l_{i}^{v}$ and $\Delta l_{i}^{r}$ represent the deformations of the initial pores and skeleton particles, respectively; and $\varepsilon_{i}^{v}$ and $\varepsilon_{i}^{r}$ represent the corresponding strains for the initial pores and the skeleton particle.

During loading, the axial strain of th e skeleton particles is expressed as:

(26) $$\varepsilon_{i}^{s}=\frac{\sigma_{i}}{E}$$

By dividing the axial stress $\sigma_{i}$ into n incremental stress levels $\Delta \sigma_{i}^{e}$(*e* = 1, 2…n) and applying the generalized Hooke’s law, the incremental pore strain (${\Delta \varepsilon_{i}}^{ve}$) can be obtained as follows:

(27) $$\sum_{e=1}^{n}\Delta \varepsilon_{i}^{ve}=\sum_{e=1}^{n}\Delta \sigma_{i}^{e}/E_{v}$$

where $E_{v}$ is the elastic modulus specifically associated with the pores.

Given that the instantaneous length of the pore portion under a stress increment $\Delta \sigma_{i}^{e}$ is $l_{i}^{ve}$, the strain is defined as:

(28) $$\varepsilon_{i}^{ve}=-\int_{l_{i}^{v\left(e-1\right)}}^{l_{i}^{ve}}\frac{dl}{l}=-\ln\frac{l_{i}^{ve}}{l_{i}^{v\left(e-1\right)}}$$

where l denotes the axial length occupied by the pores.

Integrating the relationship described in Equations (27) and (28) yields:

(29) $$\frac{\sigma_{i}}{E_{v}}=-\ln\frac{l_{n}^{v}}{l_{0}^{v}}$$

where $l_{0}^{v}$ and $l_{n}^{v}$ represent the initial and post-loading pore lengths, respectively.

This expression effectively describes the macroscopic deformation associated with pore closure. Assuming that cracks in rocks are randomly distributed, geomaterials can be idealized using a penny-shaped crack model. By introducing a weighting coefficient α to account for the pore-closure effect under axial compression, the axial strain associated with pore closure can be expressed as:

(30) $$\varepsilon_{i}^{v}=\alpha \frac{l_{i}^{v0}-l_{i}^{vn}}{l_{i}^{vo}}=\alpha \left(1-\exp\left(-\frac{\sigma_{i}}{E_{v}}\right)\right)$$

Assuming that $\frac{\sigma_{i}}{E_{v}}=\beta \frac{\sigma_{i}}{E}=\beta \varepsilon_{i}$, and substituting Equations (26) and (30) into Equation (25), the following relationship is obtained:

(31) $$\varepsilon_{i}=\alpha \left[1-\exp\left(-\beta \varepsilon \right)\right]+\frac{\sigma_{i}}{E}$$

where β is a constant determined from the experimental results.

**Figure 13:**
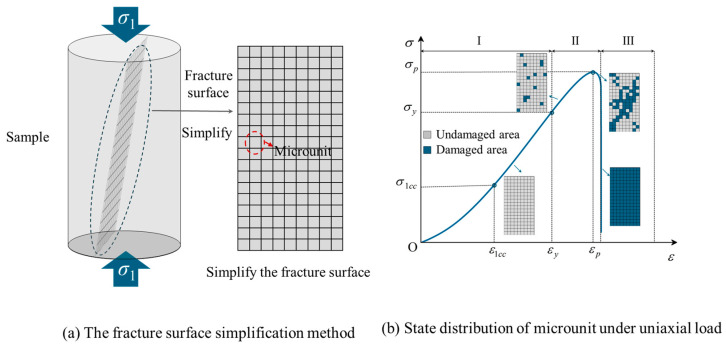
Idealized fracture-surface microunits and the corresponding stress–strain state distribution under uniaxial loading.

As shown in Figure 13, the deformation and failure process of red sandstone under loading is categorized into three stages: compaction (I), elastic (II), and failure (III), where $\sigma_{y}$ and $\varepsilon_{y}$ denote the stress and strain at the elastic limit, respectively. During the initial compaction stage, the primary internal pores close gradually under axial loading without significant crack propagation. Consequently, $D_{S}=0$, and the damage variable for red sandstone under chemical–wet–dry interaction and uniaxial compression is expressed as:

(32) $$D=\left\{\begin{array}{l} D_{CN}\;\left(0\leq \varepsilon_{1}\leq \varepsilon_{1cc}\right)\\ D_{\mathrm{CN}}+D_{S}-D_{\mathrm{CN}}D_{S\;}\;\left(\varepsilon_{1}\geq \varepsilon_{1cc}\right) \end{array}\right.$$

(2) Formulation of the Damage Constitutive Model

In damage mechanics, the effective stress $\sigma^{*}$ is defined relative to the Cauchy stress σ to characterize the constitutive behavior of damaged materials within the framework of classical elastoplastic theory. Their relationship is expressed as:

(33) $$\sigma_{i}^{*}=\frac{\sigma_{i}}{1-D}$$

The rock’s mechanical response to axial stress is governed by a constitutive model based on generalized Hooke’s law and deformation compatibility criteria:

(34) $$\varepsilon_{i}=\varepsilon_{i}^{*}=\frac{1}{E}\left[\sigma_{i}^{*}-\mu \left(\sigma_{j}^{*}+\sigma_{k}^{*}\right)\right]=\frac{\sigma_{i}-\mu \left(\sigma_{j}+\sigma_{k}\right)}{E\left(1-D_{123}\right)}$$

where $\varepsilon_{i}^{*}$ is the effective strain, $\sigma_{j}$ and $\sigma_{k}$ represent the secondary and tertiary principal stresses, respectively. This model effectively accounts for the influence of chemical–wet–dry cycles on red sandstone under axial loading:

(35) $$\sigma_{i}=\left(1-D\right)E\varepsilon_{i}+\mu \left(\sigma_{j}+\sigma_{k}\right)$$

By integrating the piecewise strain and damage variable expressions, the comprehensive damage constitutive model for red sandstone under coupled chemical–wet–dry interaction and loading is established as:

(36) $$\sigma =\left\{\begin{array}{l} E(1-D_{CN})\left\{\varepsilon -\alpha \left[1-\exp\left(-\beta \varepsilon \right)\right]\;\right\}\;\left(\varepsilon_{1}\geq \varepsilon_{1cc}\right)\\ E\left[1-(D_{\mathrm{CN}}+D_{S}-D_{\mathrm{CN}}D_{S\;})\right]\left(\varepsilon_{p}-\varepsilon_{1cc}\right)+\sigma_{1cc} \end{array}\right.$$

#### 2.3.3. Model Validation

**Figure 14:**
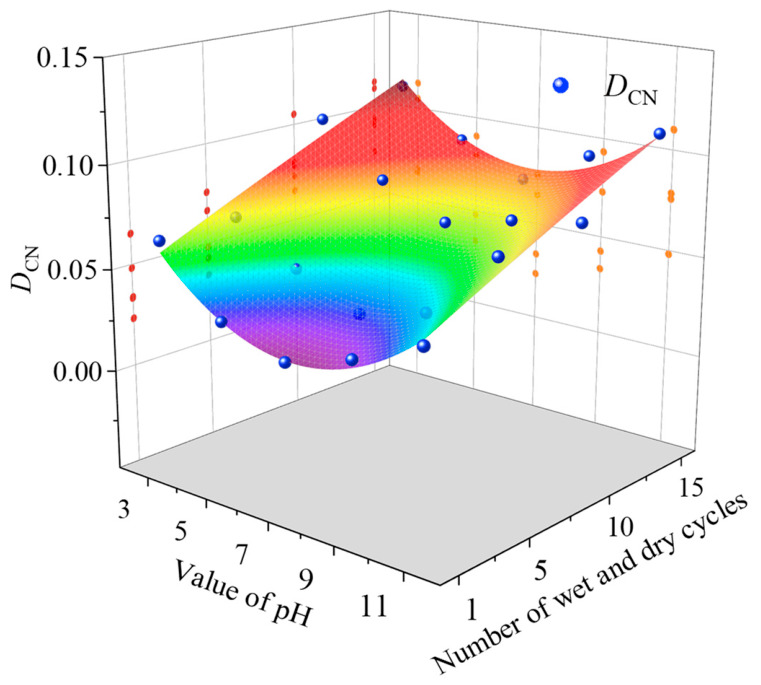
Correlation between $D_{cn}$, pH, and the number of wet–dry cycles.

Table 3 lists the parameters for the developed constitutive model. Among them, the Weibull parameter m reflects the heterogeneity of mesoscopic element strength, whereas $F_{0}$ represents the characteristic strength level. These parameters, together with the porosity-based damage variable, reflect the regulating effect of in situ hydrogels under different pH conditions. The coupled damage variable for red sandstone subjected to various chemical–wet–dry cycles, was first determined using Equation (10), with the hydrogel pore-filling effect integrated. Its sensitivity to the solution pH and the number of wet–dry cycles is graphically represented in Figure 14. A mathematical expression for the damage variable was derived through quadratic surface fitting to quantify these relationships, resulting in Equation (37). The results suggest that as the pH deviates from neutrality and the number of wet–dry cycles increases, the damage variable of red sandstone generally increases, while the in situ hydrogels formed under alkaline conditions may slightly reduce the damage increment, which is consistent with the observed mechanical degradation under chemical–wet–dry cycles.

(37) $$D_{cn}=0.12208-0.02823x+0.00442y+0.00194x^{2}-1.74749\times 10^{-5}y^{2}\;R^{2}=0.95$$

where x denotes the pH and y represents the number of wet–dry cycles.

**Figure 15:**
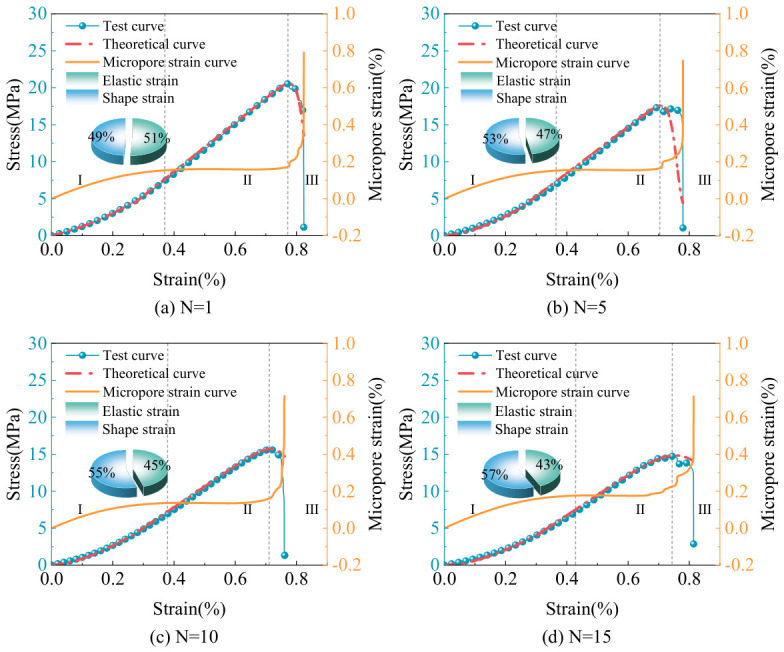
Comparison between experimental and theoretical stress–strain curves for red sandstone subjected to chemical–wet–dry cycles at pH = 3.

**Figure 16:**
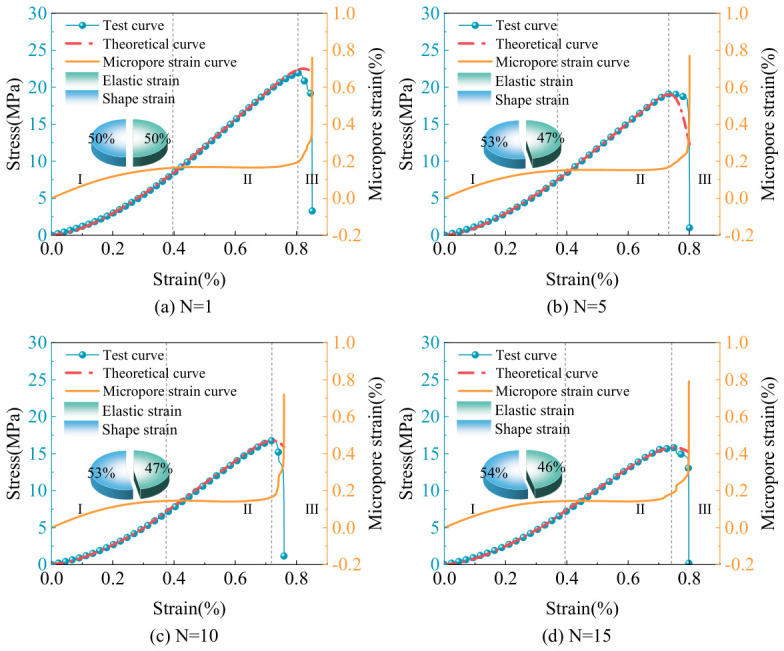
Comparison between experimental and theoretical stress–strain curves for red sandstone subjected to chemical–wet–dry cycles at pH = 5.

**Figure 17:**
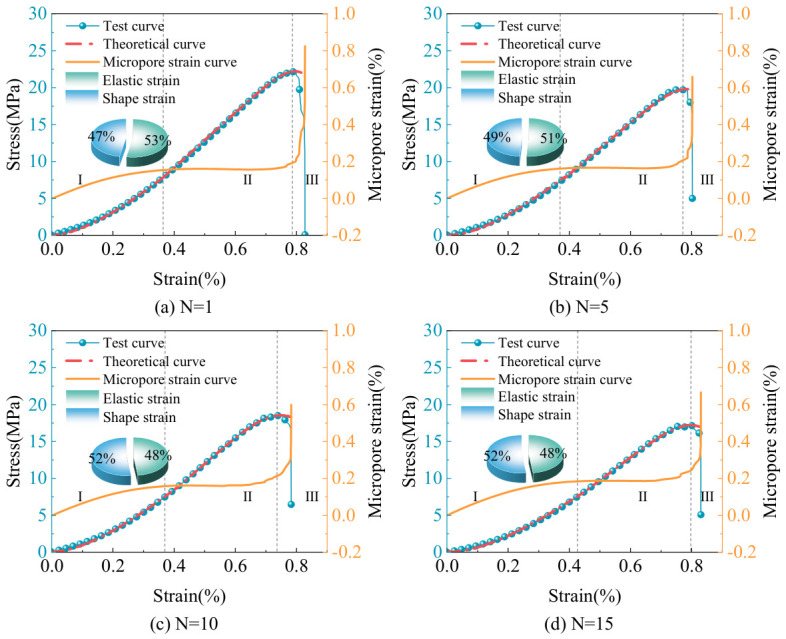
Comparison between experimental and theoretical stress–strain curves for red sandstone subjected to chemical–wet–dry cycles at pH = 7.

**Figure 18:**
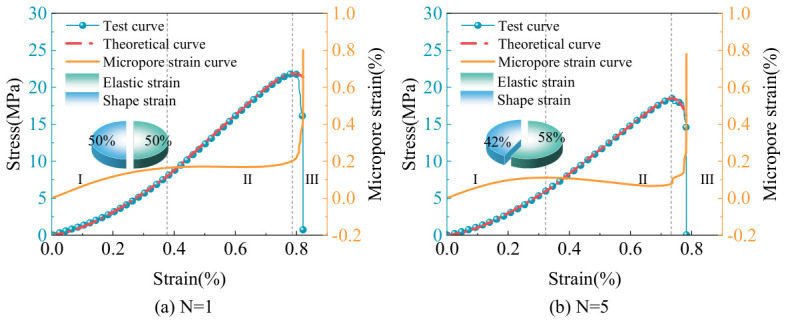

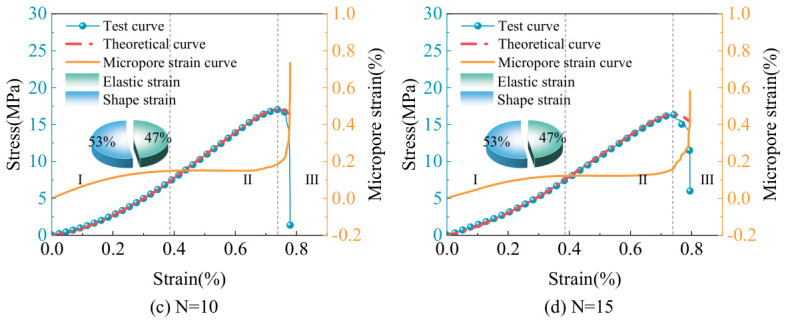
Comparison between experimental and theoretical stress–strain curves for red sandstone subjected to chemical–wet–dry cycles at pH = 9.

**Figure 19:**
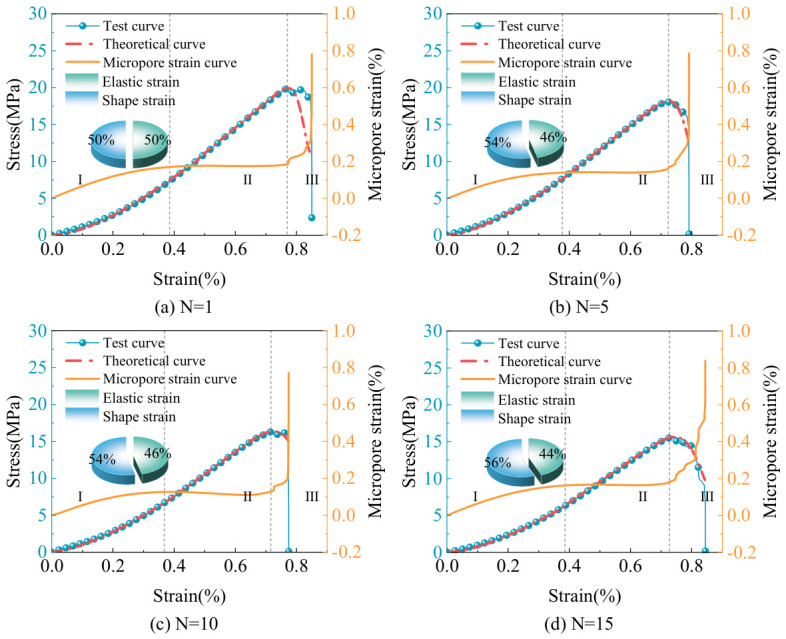
Comparison between experimental and theoretical stress–strain curves for red sandstone subjected to chemical–wet–dry cycles at pH = 11.

As illustrated in Figure 15, Figure 16, Figure 17, Figure 18 and Figure 19, the piecewise damage constitutive model presented in Equation (36), which considers hydrogel-mediated effects, effectively reproduces the stress–strain responses of red sandstone subjected to chemical–wet–dry cycles. The model results exhibit a high degree of correlation with the experimental data, effectively characterizing the mechanical behavior of the specimens after cycling, including hydrogel-induced damage inhibition under alkaline conditions.

Chemical–wet–dry cycles significantly promoted the expansion of primary pores and the propagation of internal cracks, which led to a more pronounced initial compaction phase (Stage I), while in situ hydrogels under alkaline conditions weakened this effect. This structural change increased the proportion of plastic strain and reduced elastic strain (Stage II), making the post-peak failure stage (Stage III) more prominent. Specifically, at pH = 3, as the number of cycles (*N*) increased from 5 to 10 and then to 15, the corresponding plastic strain proportions reached 50%, 55%, and 57%, respectively. These findings indicate that the proposed model can capture the complex nonlinear deformation behavior induced by the closure of pores and microcracks as the number of wet–dry cycles increases, as well as the hydrogel-mediated regulating effect. Consequently, this model serves as a useful tool for assessing the synergistic damage effects caused by various environmental and mechanical factors and provides theoretical support for the geotechnical application of in situ hydrogel materials.

### 2.4. Discussion

**Figure 20:**
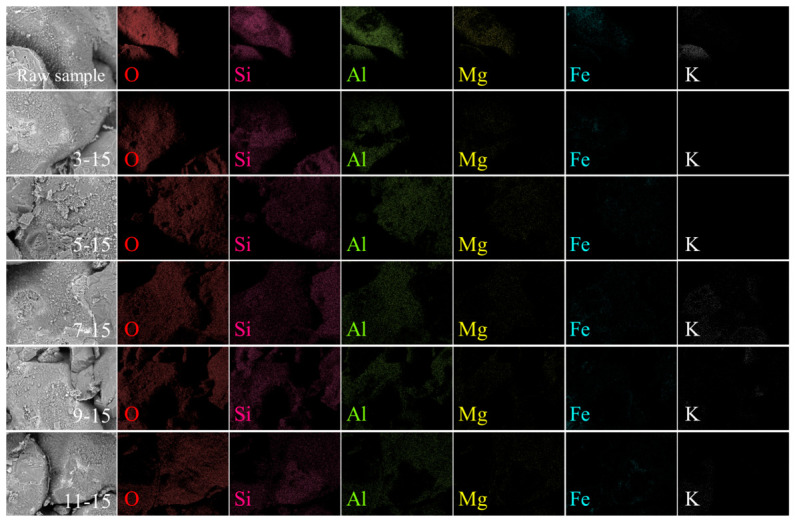
EDS mapping of elemental distribution on the red sandstone surface following chemical interaction.

The surface elemental distribution of red sandstone following chemical–wet–dry cycle interactions is illustrated in Figure 20. These elemental changes provide an important material basis for in situ hydrogel formation. Under neutral conditions (pH = 7), the relative elemental concentrations on the specimen surface exhibited the least deviation from the initial state. In this environment, only minor hydrolysis of specific hydrophilic oxides was observed, resulting in limited release of framework ions, such as Al^3+^. Furthermore, because the Si–O bonds remain relatively stable under neutral conditions, the relative Si content showed a slight increase. Although elemental migration occurred across all tested hydrochemical environments, its intensity was highly dependent on the solution pH. Under acidic conditions, the Fe signal decreased significantly, and the K signal nearly vanished. Conversely, under alkaline conditions, the spatial distributions of Si and Al became increasingly dispersed, as these elements are the key precursors for in situ hydrogel formation.

**Figure 21:**
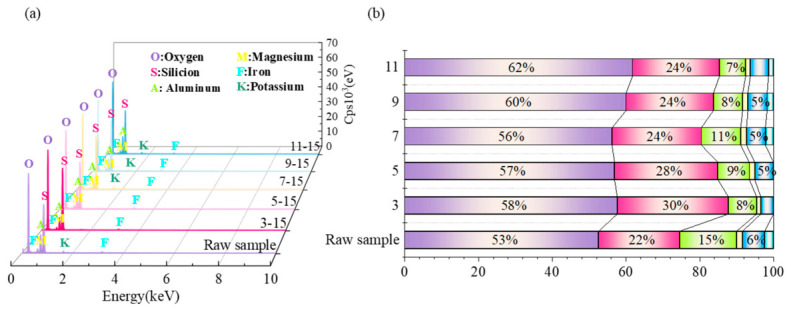
Quantitative EDS analysis of red sandstone samples: (**a**) EDS spectra; (**b**) elemental atomic percentages.

Figure 21 presents the EDS spectra and the corresponding atomic percentages on the sample surface after chemical–wet–dry interactions. The results show that O, Si, Al, Fe, K, and Mg were the most abundant elements detected, which are closely associated with the elemental composition of in situ hydrogels. Given that the red sandstone is primarily composed of quartz and clay minerals, the combined proportions of O and Si exceeded 78%. Following chemical corrosion, the proportions of Al, Fe, K, and Mg decreased to varying degrees, which is consistent with the XRD results. Under acidic conditions, the proportion of K approached zero, primarily because potassium feldspar is highly sensitive to H^+^ and dissolves rapidly in acidic environments. In alkaline environments, the dissolution of clay minerals, combined with the detachment of dolomite and quartz, resulted in a measurable reduction in Si and Al content [17]. These released Si and Al ions provide the key building blocks for the three-dimensional network of in situ hydrogels.

**Figure 22:**
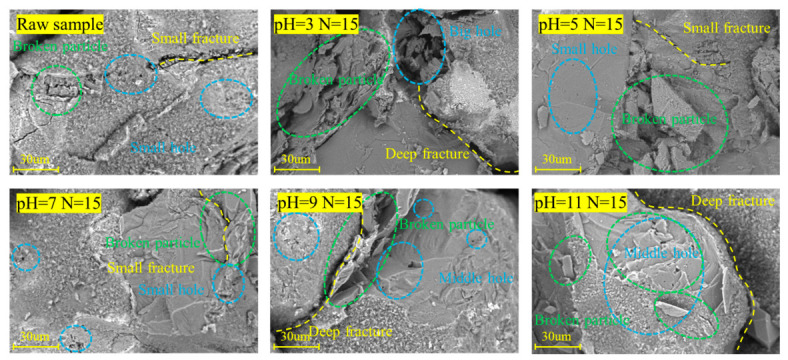
SEM micrographs showing microstructural deterioration and crack propagation at 2000× magnification.

The surface micromorphological features of the specimens after undergoing chemical–wet–dry cycle interaction are depicted in Figure 22. These microstructural changes also provide space for the growth of in situ hydrogels. In the original state, the specimen surface was generally smooth, characterized by only a few fine microcracks and isolated, tiny pores. However, after chemical–wet–dry cycling, the number of surface micropores increased markedly, and larger cracks and voids gradually developed. These evolving pores and fractures provide sufficient space for the nucleation and growth of in situ hydrogels. Concurrently, the cementation between mineral particles weakened noticeably.

The distinct erosive impacts of various hydrochemical solutions on the rock’s microstructure were clearly evident. Specifically, acidic reactions facilitated the dissolution of mineral constituents, triggering the formation of new voids. This process led to increased surface roughness and a noticeably more porous, open internal architecture. Furthermore, mineral grain boundaries became increasingly distinct, and pores expanded and exhibited enhanced connectivity; consequently, both microcracks and pores grew significantly larger than those observed in the pristine samples [46]. Under alkaline conditions, the reaction products formed continuous in situ hydrogels within the rock pores and fractures. These hydrogels filled the pore space, adhered to mineral surfaces, and partially hindered crack propagation. However, this mitigating effect was ultimately insufficient to compensate for the overall mechanical deterioration driven by mineral loss and internal structural damage [47]. Finally, the development of these new pores established fresh seepage channels, which in turn accelerated water–rock interactions and further promoted the in situ formation and distribution of hydrogels, while contributing to the long-term degradation of the macroscopic mechanical properties of the red sandstone.

Following the uniaxial compressive tests, the fracture surfaces of the chemically corroded specimens appeared relatively smooth. Two distinct failure modes were observed: transgranular and intergranular fractures, both accompanied by extensive mineral spalling. The structurally weak planes formed by chemical action were clearly exposed, and this feature was particularly evident under acidic conditions. Under alkaline conditions, the in situ hydrogels formed under alkaline conditions altered the crack propagation path, further indicating their damage-regulating effect. From a mechanical perspective, the interaction between hydrochemical solutions and wet–dry cycles induced physicochemical reactions that weakened interparticle cementation and increased particle spacing. These synergistic effects generated numerous structural weak interfaces, thereby reducing the total energy required for failure and increasing the probability of intergranular fractures.

## 3. Conclusions

(1) Under the synergistic interaction of chemical environments and wet–dry cycles, the mechanical parameters of red sandstone show distinct nonlinear degradation. The severity of this deterioration follows the order of acidic > alkaline > neutral and increases with the number of wet–dry cycles. The weaker degradation in alkaline environments is closely related to the inhibitory effect of in situ formed hydrogels. After 15 cycles at pH = 3, the deterioration degrees of peak strength and elastic modulus reach 38.21% and 27.12%, respectively, accompanied by a consistent reduction in P-wave velocity.

(2) Mercury intrusion tests show that the cumulative mercury intrusion volume increases steadily with the number of wet–dry cycles, indicating a general rise in porosity. At pH = 7, porosity increases from 21.51% in the natural state to 23.14%, where the porosity increments reach 3.00% and 2.86% at pH = 3 and pH = 11, respectively. The slightly lower porosity increment under alkaline conditions is attributed to the pore-filling effect of in situ hydrogels. In acidic environments, the most probable pore diameter shifts from 21.32 μm toward larger pore sizes, accompanied by enhanced pore connectivity.

(3) Ion concentration measurements demonstrate that the concentrations of Ca^2+^ and K^+^ decrease as the number of wet–dry cycles increases. Specifically, at pH = 3, the Ca^2+^ concentration decreases by approximately 52–63% relative to its initial value. At pH = 7, the concentration gradually decreases from 3.225 mmol/L to 0.957 mmol/L. EDS analysis shows that the K content under acidic conditions approaches zero, indicating substantial dissolution of potassium feldspar, which is consistent with the XRD findings. The dissolution of these mineral ions provides an important material basis for in situ hydrogel formation in alkaline environments.

(4) SEM observations confirm that acidic conditions promote rapid pore development, with pores enlarging and gradually becoming interconnected. Under alkaline conditions, the reaction products form in situ hydrogels within the rock matrix. These hydrogels partially inhibit crack propagation. Ultimately, the combined effects of crack development and pore expansion significantly reduce the effective load-bearing area of the rock.

(5) The statistical damage constitutive model developed in this study, incorporating the hydrogel-mediated damage regulation effect, effectively represents the nonlinear stress–strain behavior of red sandstone. It reasonably captures damage evolution under chemical–wet–dry cycle interaction and external loading. This is demonstrated by the strong agreement between the experimental and theoretical curves. This model provides a useful framework for assessing multi-factor damage effects and supports the practical application of in situ hydrogel materials in geotechnical engineering.

## 4. Materials and Methods

### 4.1. Sample Preparation

**Figure 23:**
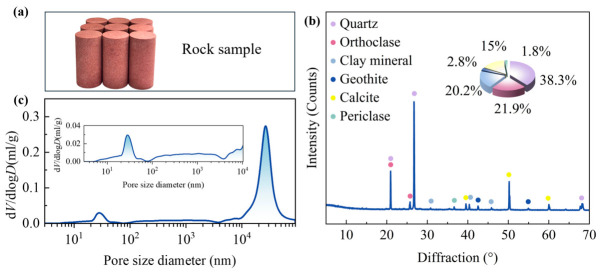
Characteristics of the tested red sandstone samples: (**a**) rock samples; (**b**) XRD pattern and mineral composition; (**c**) pore-size distribution from MIP tests.

The soft red sandstone specimens used in this study were obtained from a typical red-bed soft rock formation. Sample preparation strictly followed the guidelines established by the International Society for Rock Mechanics (ISRM) [48]. The rock mass was processed into standard cylindrical specimens measuring 50 mm × 100 mm (D × H). The resulting samples displayed a uniform red coloration and were devoid of any macroscopically visible defects or joints. Initial mechanical and physical characterization of the intact rock revealed a uniaxial compressive strength, elastic modulus, average density, and porosity values of 24 MPa, 3.89 GPa, 2010 kg/m^3^, and 21.51%, respectively, as shown in Figure 23b. Mineralogical analysis via XRD indicated that the red sandstone was primarily composed of quartz (38.2%), with secondary constituents including feldspar (21.8%) and clay minerals (20.2%), along with additional constituents including calcite (15.0%), goethite (2.8%), and dolomite (1.8%).

### 4.2. Experimental Scheme

Red-bed soft rock masses are commonly developed in hydrogeological environments where alkaline groundwater may occur [49]. Furthermore, the field environment may be affected by long-term acid rain erosion and periodic fluctuations in groundwater and river levels, placing the rock mass in a complex, dynamically evolving hydrochemical environment.

**Figure 24:**
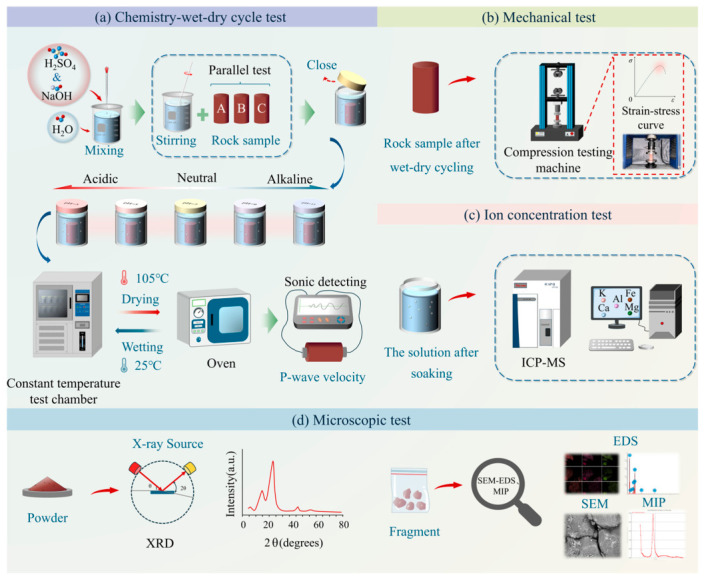
(**a**–**d**) Schematic flowchart of the experimental procedure, including sample treatment, mechanical testing, and microstructural characterization.

(1) Under natural conditions, the chemical corrosion of rock is a long-term evolutionary process governed by varying intensities of acidic or alkaline environments. This process is further influenced by the heterogeneous distribution of groundwater seepage and the temporal and spatial variability of water pollution. The experimental conditions designed to simulate these hydrochemical processes, involving varying pH levels and wet–dry cycles, are detailed in Table 4.

(2) Solutions with different pH values were prepared by adjusting the concentrations of sulfuric acid (H_2_SO_4_) and sodium hydroxide (NaOH). Specimens were immersed in these solutions for static soaking at a constant temperature of 25 °C, as shown in Figure 24a. To maintain a stable chemical environment, freshly prepared solutions were replaced at the start of each wet–dry cycle to keep the pH approximately constant. A single wet–dry cycle comprised 24 h of immersion followed by 24 h of drying in a constant-temperature oven at 105 °C. Identical drying treatments were applied to all samples to ensure moisture consistency and minimize moisture-related effects on subsequent test results.

(3) Following the chemical–wet–dry cycle treatments, the P-wave velocities of the samples were measured using an ultrasonic rock parameter tester (BJZJ-YTCS-2506181, Beijing Zhongjiao Jianyi Technology Development Co., Ltd., Beijing, China) Uniaxial compression tests were subsequently performed using a computer-controlled electronic universal testing machine (Shenzhen SUNS Technology Stock Co., Ltd., Shenzhen, China) with a maximum capacity of 300 kN, at a loading rate of 0.05 mm/min, as shown in Figure 24b. A total of 190 rock specimens were tested to ensure statistical reliability.

(4) Finally, microstructural characterization was conducted following the uniaxial compressive strength tests. XRD (D8 ADVANCE, Bruker AXS LLC, Madison, WI, USA) and ICP-MS (Thermo Fisher Scientific, Waltham, MA, USA) were used to analyze mineralogical alterations and ionic interactions during water–rock reactions. MIP (AutoPore V 9600, Micromeritics Instrument Corporation, Norcross, GA, USA; MicroActive AutoPore V 9600 software, version 2.03.00) was employed to evaluate shifts in pore-size distribution and morphology resulting from the chemical–wet–dry cycles. SEM-EDS (Phenom XL, Phenom-World B.V., Eindhoven, The Netherlands) was used to investigate the microstructural changes and elemental distribution within the rock specimens (Figure 24c,d).

## Figures and Tables

**Table 1:** Mechanical parameters of red sandstone under various chemical–wet–dry scenarios.

pH	Number of Wet–Dry Cycles (*N*)	$\sigma_{c}$ (MPa)	E (GPa)	$\varepsilon_{c}$ (%)
3	1	20.58	3.42	0.78
	5	17.64	3.26	0.71
	10	15.70	2.85	0.72
	15	14.83	2.84	0.75
5	1	21.95	3.60	0.80
	5	19.18	3.39	0.73
	10	16.79	3.03	0.72
	15	15.82	2.94	0.75
7	1	22.22	3.73	0.79
	5	19.80	3.53	0.78
	10	18.54	3.49	0.75
	15	17.20	3.08	0.80
9	1	21.86	3.65	0.79
	5	18.50	3.26	0.73
	10	17.56	3.08	0.74
	15	16.35	2.92	0.77
11	1	19.95	3.41	0.77
	5	18.20	3.23	0.72
	10	16.39	3.02	0.72
	15	15.64	2.84	0.74

**Table 2:** Quantitative porosity variations in red sandstone across different experimental conditions (%).

Raw Sample	*N*	pH				
		3	5	7	9	11
21.51	1	22.81	22.00	21.79	21.99	22.34
	5	22.86	22.34	22.00	22.20	23.15
	10	24.19	23.29	22.82	23.06	23.24
	15	24.51	23.64	23.14	23.75	24.37

**Table 3:** Identified parameters for the statistical damage constitutive model.

pH	Number of Dry and Wet Cycles (*N*)	m	$F_{0}$	α	β	$R^{2}$
3	1	34.36	18.50	0.19	4.31	0.99
	5	31.65	15.91	0.18	5.16	0.92
	10	12.53	15.42	0.14	6.45	0.99
	15	7.032	16.20	0.22	3.93	0.99
5	1	12.82	18.50	0.19	4.78	0.99
	5	16.13	13.01	0.17	5.51	0.99
	10	13.62	15.57	0.17	5.03	0.99
	15	7.48	15.67	0.16	6.07	0.99
7	1	9.01	20.46	0.17	5.32	0.99
	5	6.8	18.53	0.18	5.56	0.99
	10	4.55	16.84	0.18	5.24	0.99
	15	5.88	14.94	0.20	4.88	0.99
9	1	9.38	19.00	0.21	4.14	0.99
	5	16.13	14.65	0.17	5.09	0.99
	10	9.26	14.45	0.17	5.16	0.99
	15	12.20	16.03	0.14	5.01	0.99
11	1	17.54	11.86	0.19	4.78	0.98
	5	14.86	12.98	0.15	5.63	0.99
	10	9.17	12.00	0.17	4.98	0.99
	15	11.19	13.01	0.18	4.94	0.99

**Table 4:** Summary of experimental conditions detailing variations in hydrochemical pH levels and the number.

Variable	Condition
Number of wet-dry cycles (*N*)	1, 5, 10, and 15
Value of pH	3, 5, 7, 9, and 11

## Data Availability

Data are contained within the article.
